# School-Based Intervention Programs: Conceptual, Empirical, Practical, and Equity-Focused Considerations Across Development, Implementation, Evaluation, and Dissemination

**DOI:** 10.3390/bs16101789

**Published:** 2026-10-01

**Authors:** John C. Begeny, Marisa E. Marraccini

**Affiliations:** 1 Department of Psychology, North Carolina State University, Raleigh, NC 27695, USA; 2 School of Education, University of North Carolina at Chapel Hill, Chapel Hill, NC 27599, USA; mmarracc@unc.edu

**Keywords:** school-based intervention programs, intervention development, implementation science, program evaluation, dissemination, intervention adoption, professional judgment, educational equity

## Abstract

School-based intervention programs play an essential role in promoting students’ academic, behavioral, and social–emotional development. Achieving meaningful and equitable outcomes, however, depends on more than the availability of intervention programs. It also requires high-quality intervention work across the interconnected domains of intervention development, implementation, evaluation, and dissemination. Substantial scholarship exists within each domain, yet relevant concepts, considerations, and practical guidance are often distributed across separate studies and frameworks, making it difficult for intervention scientists and intervention adopters to develop an integrated understanding of intervention work. This paper provides a functional conceptualization of intervention work by synthesizing foundational concepts, empirical and practical considerations, and guiding questions across its four primary domains. Rather than proposing a new intervention framework, the paper complements existing frameworks by clarifying what each domain is intended to accomplish, the knowledge and products it generates, and how activities within one domain can inform and improve decisions made throughout the others. Collectively, the paper argues that high-quality intervention work depends not only on excellence within each domain, but also on intentionally designing activities within each domain so that the resulting knowledge and resources contribute to the broader system of intervention research and practice. The paper is written for two primary audiences: (a) intervention scientists engaged in developing, implementing, evaluating, or disseminating intervention programs, and (b) intervention adopters responsible for selecting, implementing, evaluating, and making continued-use decisions about programs in educational settings. Three primary contributions are provided. First, the paper clarifies foundational concepts, essential considerations, and key questions associated with each domain. Second, it illustrates how the four domains collectively contribute to the development, implementation, evaluation, dissemination, adoption, and sustained use of high-quality intervention programs. Third, it translates this conceptual perspective into practical guidance through tables, 12 key questions and considerations, and separate Reflection Guides designed to support professional judgment, high-quality intervention science, and evidence-driven intervention adoption and use.

## 1. Introduction

Schools play a critical role in fostering students’ academic, social, and emotional development, making effective intervention programs a cornerstone of educational practice. Despite substantial advances in educational research, many students continue to experience learning or behavioral challenges that could be better addressed through broader access to evidence-based interventions (National Center for Education Statistics, 2024; OECD, 2023, 2024). Meeting this need depends not only on expanding the number of high-quality intervention programs available, but also on ensuring these programs are intentionally developed, successfully implemented, rigorously evaluated, appropriately disseminated, and sustained across diverse educational contexts.

Achieving these goals, however, is far from straightforward. Intervention work—from the initial conception of an intervention program through its development; implementation; evaluation; dissemination; adoption; continued use; and, when warranted, refinement—is inherently complex (Moore et al., 2019; Nilsen, 2024; Wanzer, 2021). For example, interventions must be grounded in scientific rigor while remaining feasible and acceptable in real-world settings that often face competing priorities, limited resources, and diverse needs of intervention beneficiaries (Begeny et al., 2023; Neshteruk et al., 2021; Onken, 2022). Moreover, the complexity of human behavior and educational systems makes it difficult to identify which intervention components are most responsible for positive outcomes, sustain those outcomes over time, and balance fidelity to the original intervention design with possible adaptations that may be necessary to fit specific educational contexts (Collins, 2018).

Although definitions and descriptions of intervention work differ in the scholarly literature—both across and within disciplines—there are arguably four primary domains that must be considered in the context of intervention programs: development, implementation, evaluation, and dissemination. There are several existing models and frameworks that have been developed and used to inform the “how to” within some of the four primary domains (e.g., Bartholomew et al., 1998; Damschroder et al., 2009; Glasgow et al., 1999; Johnson et al., 2025; Michie et al., 2011), and approaches have been developed and used in several countries around the globe (Nilsen, 2024; Onken et al., 2014; Skivington et al., 2021; World Health Organization, 2010). Professionals focused on public health and medicine have arguably produced the most work on frameworks for navigating the intervention life cycle (i.e., the structured, iterative process used to design, implement, evaluate, and scale an intervention), but models designed specifically for school-based intervention or prevention are also available (e.g., Institute of Education Sciences [IES] & National Science Foundation [NSF], 2013; McBride, 2016).^1^

Existing frameworks differ in scope, intended audience, and purpose. Collectively, they provide valuable guidance regarding how to conduct various aspects of intervention work.^2^ However, because these frameworks typically emphasize particular phases of the intervention life cycle or specific methodological approaches, readers often need to consult multiple resources to develop a broader understanding of intervention work as a whole (Fort et al., 2023; King et al., 2020; Reilly et al., 2020). This fragmentation may also be reinforced by disciplinary boundaries, differing professional roles and priorities, resource constraints, and institutional structures that shape how intervention work is developed, studied, supported, and disseminated. As a result, although considerable scholarship exists within each of the four domains of intervention work, the broader conceptual connections among them—and the ways they collectively contribute to high-quality intervention work—are often less explicit.

This paper does not propose a new framework for developing, implementing, evaluating, or disseminating intervention programs. Rather, it offers a functional conceptualization of intervention work. As used in this paper, a functional conceptualization refers to an integrative way of organizing and connecting distinct domains of intervention work according to the purposes they serve, the knowledge and products they generate, and how each can inform and strengthen work within the other domains. Thus, rather than prescribing a particular process for conducting intervention work, we seek to clarify the functions and interconnections of these domains as well as key considerations across the intervention life cycle. In doing so, we aim to help intervention scientists and intervention adopters develop a shared understanding of intervention work while strengthening their ability to plan, evaluate, and improve intervention efforts. Our overall goal is to provide a resource that complements existing frameworks and supports informed professional judgment throughout the intervention life cycle.

In developing this conceptualization, we draw on both the broader scholarly literature and our applied experiences with intervention development, implementation, evaluation, and dissemination. The first author’s perspective has been shaped by more than two decades of leading the development, implementation, evaluation, and dissemination of multiple school-based academic intervention programs (e.g., Begeny et al., 2010; Begeny et al., 2018; Begeny et al., 2020) and implementation models (e.g., Begeny et al., 2012; Begeny et al., 2023), including organizational efforts to advance equitable adoption and sustained use of research-supported interventions. The second author brings complementary experience from the development and dissemination of practical guidance for youth hospitalized for suicide-related crises (e.g., Marraccini et al., 2024b) and the development and evaluation of Practice Experiences for School Reintegration (PrESR), a virtual reality intervention designed to help hospitalized adolescents prepare to return to school following discharge (e.g., Marraccini et al., 2024a; Marraccini et al., 2026). These and related experiences inform the practical perspective reflected throughout the paper. By outlining foundational concepts, practical insights, empirical considerations, illustrative examples, and guiding questions across the four interconnected domains, our aim is to support high-quality, practically consequential, and equity-focused intervention work.

### 1.1. Intended Audience and Specific Goals of This Paper

The intended audience for this paper includes individuals interested in understanding the key concepts, considerations, and linkages associated with developing, implementing, evaluating, and disseminating school-based interventions. However, this paper is written with two primary audiences in mind: intervention scientists and intervention adopters. Throughout this paper, *intervention scientists* refer to researchers engaged in (or aspiring to engage in) one or more aspects of intervention development, implementation, evaluation, or dissemination. We use the term *intervention adopters* to refer to individuals or teams responsible for selecting whether to adopt and use school-based interventions within a particular educational context. These may include classroom educators, school principals, district curriculum directors, Multi-Tiered Systems of Support (MTSS) leadership teams, afterschool program directors, district-level committees, or support staff.

A central purpose of this paper is to integrate fragmented scholarship across four interconnected domains of intervention work (development, implementation, evaluation, and dissemination) into a functional conceptualization and a practical set of resources intended to strengthen (a) the production of high-quality school-based intervention programs, and (b) the professional judgments involved in their adoption and use. More specifically, this paper has three primary goals and intended contributions. First, we seek to clarify foundational concepts, essential considerations, and key questions within each of the four domains of intervention work. Although extensive scholarship has examined specific components of intervention work, this paper integrates these complementary bodies of scholarship into a functional conceptualization of intervention work that emphasizes not only what occurs within each domain, but also what each domain is intended to accomplish, the knowledge and products it generates, and how it contributes to the other domains.

Second, we highlight the interdependent nature of the four domains and emphasize connections that are often overlooked when domains are considered separately or in ways that implicitly prioritize one or two domains over others. Decisions related to intervention development influence implementation demands, evaluation approaches, dissemination potential, and eventual adoption. Similarly, considerations related to implementation, evaluation, dissemination, adoption, scalability, and sustainability should inform intervention decisions from the earliest stages of development. More broadly, we contend that activities within each domain should be designed not only to accomplish the goals of that domain, but also to support and strengthen work occurring within the other domains. Recognizing these interconnections is critical for producing intervention programs that are not only effective, but also feasible, usable, sustainable, and equitable across diverse educational contexts.

Third, we offer practical questions, recommendations, and tools to support decision-making by both intervention scientists and intervention adopters. For intervention scientists, these resources are intended to support planning and reflection throughout intervention work by identifying important considerations that may strengthen intervention quality and long-term impact. For intervention adopters, these resources are intended to support evaluation and selection of intervention programs by highlighting characteristics of high-quality interventions that may influence their likelihood of benefiting the students and communities they serve. User-friendly tables and Reflection Guides are provided to facilitate practical application of this paper as a scholarly resource.

One implication of the conceptual perspective presented throughout this paper is that intervention development extends beyond the creation of an effective intervention program. High-quality intervention programs should equip program users with practical guidance—grounded in evidence and accumulated experience—that helps them address the practical implementation challenges they may encounter in real-world/authentic educational settings, including when and how adaptations may be appropriate, how implementation fidelity can be supported and monitored,^3^ and how ongoing local program evaluation can be conducted in ways that are both meaningful and feasible.

## 2. Defining Intervention and Establishing the Scope of This Paper

Before discussing the four primary domains of intervention work, we first clarify several foundational concepts that shape how intervention efforts are conceptualized throughout the paper. Specifically, we define what we mean by “intervention,” distinguish intervention programs from intervention strategies, and describe why intervention program manuals represent one of the primary products of intervention development and an essential resource across the four domains of intervention work.

### 2.1. What Is an Intervention?

Although “intervention” is widely used across psychology, education, public health, and related fields, the term has multiple meanings depending on disciplinary context. For example, definitions may emphasize therapeutic approaches, experimental manipulations, prevention efforts, or broader attempts to alter conditions and improve functioning (e.g., American Psychological Association, n.d.; Merriam-Webster, n.d.). For the purposes of this paper, we use the definition of intervention provided in the book, *On Understanding Intervention in Psychology and Education* (Adelman & Taylor, 1994). The authors contend that a definition of intervention should have some specificity, but also be “broad enough to reflect the full nature and scope of intervention means and ends” (p. 41)—and they provide a definition that is well-suited for the focus of this paper:

Intentional intervention aims at producing intended outcomes through planned processes. The intended outcomes encompass maintenance, change (development, improvement), or transformation with respect to problematic or nonproblematic conditions of systems (person, environment, or both). Besides planned processes, unplanned transactions occur. The combined processes may or may not produce intended outcomes, and may produce unintended outcomes; also some outcomes may be negative.

(pp. 41–42)

Three aspects of this definition are particularly relevant to the present paper. First, decisions made throughout intervention work influence activities within the other domains. For example, decisions made during intervention development influence implementation demands, evaluation approaches, dissemination potential, and ultimately the likelihood that an intervention will benefit intended recipients. Second, intervention ought to be intentional, including the processes of developing, implementing, evaluating, disseminating, and scaling an intervention program. Third, interventions are intended to create a positive outcome (e.g., behavior change, academic learning, maintenance of desired behavior, etc.), but in the process of providing intervention, unintended or negative outcomes may emerge—and no one intervention, even if shown to be effective in rigorous research, will be beneficial for all intended beneficiaries. This final point is especially important because intervention scientists and intervention adopters must acknowledge that even effective interventions may produce unintended consequences or fail to benefit all intended recipients (Conaway et al., 2024; Richardson, 2018; Williams et al., 2022), and these realities must also be considered in designing evaluation and efforts to promote educational equity.

### 2.2. What Is an Intervention Program Compared to an Intervention Strategy, and What Are Some Relative Advantages of Intervention Programs?

In education and psychology, intervention programs are typically distinguished from intervention strategies (Begeny et al., 2012; Durlak et al., 2011; Lee et al., 2026; Mendez, 2016; Newson et al., 2025). Although both can be beneficial, distinctions between them can sometimes be blurred (Coolong-Chaffin et al., 2024). Based on the existing scholarly literature and our applied intervention work, the following sections highlight the primary distinctions.

#### 2.2.1. Intervention Strategies

Intervention strategies are individual techniques or instructional practices that may support student learning or behavior, but they tend to be more flexible (less structured) in their implementation and do not constitute a formal intervention unless (a) implementation procedures and necessary materials are clear, well documented, and used consistently across students and implementers; (b) they are systematically used to target a specific type of student need; and (c) it is clear how the strategy can be systematically monitored for effects and delivered with fidelity. Joseph (2024) offers a good example of a well-developed intervention strategy referred to as word boxes—which is designed to help early readers connect the phonological properties of words to spelling patterns—but there are certainly others.

Many intervention strategies (e.g., repeated readings to support literacy; classroom token economies or other reward systems to increase desired student behavior; schema instruction to improve word problem solving in math) are described in practitioner-focused materials such as those available online or within the scholarly literature on school-based intervention studies. When there is clear research evidence of positive effects for a respective intervention strategy, it could potentially be useful for school-based practice or for the future development of an intervention program; however, intervention adopters and potential users of intervention strategies should consider common disadvantages of using a strategy. To illustrate with just a few examples, it may be unclear how to effectively use strategies for students with varying needs or across different grade levels; appropriate implementation materials may not be readily available; and implementation steps, acceptable modifications, implementation fidelity standards, or guidance regarding ongoing evaluation in authentic educational settings are often limited or unclear.

#### 2.2.2. Intervention Programs

Comparatively, we define a school-based intervention program as a structured and intentionally developed approach that: (a) targets clearly specified goals and intended beneficiaries; (b) integrates one or more evidence-based strategies into a coherent approach; (c) has undergone systematic development and evaluation; (d) provides users with the implementation materials needed to deliver the intervention (e.g., curricula, workbooks, activity sheets, tracking forms, or related materials) and, when appropriate, protocols to support consistent delivery and implementation fidelity; and (e) includes a detailed intervention manual that not only describes the essential ways to use the program but also provides practical guidance—grounded in accumulated evidence and experience—to help program users implement the intervention successfully in a range of real-world educational settings (e.g., guidance related to implementation fidelity standards, acceptable adaptations, ongoing local program evaluation, and addressing practical implementation challenges that may arise).

Collectively, intervention strategies are not inherently less valuable than intervention programs; rather, they generally reflect a different level of specificity and structure. A strategy may be an important component of an intervention program, but by itself it may not provide sufficient efficacy or it may lack proper implementation guidance (e.g., it may be unclear who should receive the intervention, how it should be implemented, what resources are needed, how outcomes should be monitored, or how to navigate practical challenges that may emerge). Well-developed intervention programs also reflect knowledge generated across the four domains of intervention work because there is a more central focus on purpose and evidence (e.g., clear goals, intended beneficiaries, intentional development and evaluation), implementation supports (e.g., structured approaches, implementation guidance, and materials), as well as replication and sustainability (e.g., providing detailed documentation and practical guidance within an intervention manual). In this paper, we focus our attention on intervention programs given their relative benefits.

In many educational contexts, with the growing use of tiered support systems such as MTSS, intervention adopters sometimes seek to adopt “standard protocol” intervention programs that involve educators implementing the same structured program with all or most students in a school or district who present with a specified difficulty; alternatively, some adopters may be seeking an intervention for one or a small number of students for whom a more individualized “problem-solving” approach is most optimal (for extended discussion, see Begeny et al. (2012) or Zhang et al. (2023)). The key considerations and Reflection Guide for Intervention Adopters described later in this paper can be used by adopters considering both standard protocol intervention programs and those adopted for individualized problem-solving.

Of course, not all intervention programs are equally beneficial, and distinguishing among their relative advantages and disadvantages—including the contexts for which they are intended—can often be challenging for intervention adopters. Assessments about the appropriateness of a particular program should be made in relation to what it is designed to accomplish and, when possible, in comparison with programs addressing similar educational needs and intended recipients rather than with all school-based interventions. For example, the relevant considerations may differ in an area where many programs are available, such as phonics interventions for early elementary students, compared with an area where relatively few programs exist, such as interventions designed to improve critical thinking among high school students. Throughout subsequent sections, we present additional questions and considerations to help intervention scientists and intervention adopters examine intervention development, implementation, evaluation, and dissemination. Before turning to an overview of these four primary domains, however, it is important to discuss intervention program manuals and their value across intervention work.

#### 2.2.3. Intervention Program Manuals

Intervention manuals—sometimes referred to as teacher manuals, instructional manuals, user manuals, guidebooks, or similar terms—are among the most critical products of intervention development and are essential resources for both intervention users and intervention adopters. Intervention manuals represent a particularly important example of how the four domains of intervention work intersect. A well-developed manual illustrates how knowledge generated during intervention development, implementation, evaluation, and dissemination can be translated into practical guidance grounded in accumulated evidence and experience.

Depending on the type of intervention, program manuals may differ in format and scope, address somewhat different topics, and be written for different audiences (e.g., highly technical manuals intended for researchers or specialized practitioners, manuals written for a broad audience of educational practitioners, or combinations of these approaches). We generally encourage those writing intervention manuals to be mindful of all potential audiences, but we do not recommend one style over another, provided the content is clear, well organized, and intentional.

Regardless of their format, we believe there are many specific topics that manuals for school-based interventions should address because the intervention manual is one of the primary mechanisms through which knowledge generated across intervention work becomes accessible to future program users and adopters. Although the appropriate content will vary according to the intervention, a sufficiently comprehensive manual should at least address the following about the program: (a) its purpose, supporting evidence, and relevant limitations; (b) its intended recipients and users; (c) its core intervention components, implementation procedures, and permissible adaptations; and (d) practical guidance concerning materials, training, coaching, fidelity, implementation challenges, and local evaluation. Such guidance can assist intervention scientists who are developing or revising programs while also helping intervention adopters determine whether a program provides sufficient information and support for high-quality implementation. When not provided through separate companion resources, the manual should also include the implementation materials needed to use the program, such as activity sheets, tracking forms, curricula, or related materials. With these foundational concepts established, we next turn to an overview of the four primary domains of intervention work.

## 3. Overview of the Four Primary Domains of Intervention Work

Earlier we identified the four primary domains of intervention work as development, implementation, evaluation, and dissemination. This four-domain organization was not derived through a formal systematic review or consensus process. Rather, it reflects an organizing conceptualization that developed through the first author’s intervention work over many years and has been informed by scholarship across several areas, such as iterative design, implementation science, program evaluation, research design, and dissemination science. Although existing frameworks organize intervention work in various ways, the four domains discussed in this paper represent broad functions reflected across the intervention literature. Notably, they are also highly consistent with a recent systematic effort to synthesize public health intervention frameworks (Muellmann et al., 2026).

The four domains are therefore conceptualized as broad and overlapping, rather than an exhaustive taxonomy or a sequence of stages. Each encompasses a range of both overlapping and distinct activities, concepts, and scholarly traditions that warrant initial clarification. Understanding both the unique contributions of each domain and their interrelationships provides the conceptual foundation for the remainder of this paper. Because this paper is a conceptual synthesis rather than a systematic review, no formal search or selection protocol was used; instead, the scholarship and frameworks incorporated throughout were selected purposively to represent foundational and relevant work across intervention development, implementation, evaluation, and dissemination.

### 3.1. Intervention Program Development

Intervention development is the foundation upon which all subsequent intervention work depends. By development, we refer to the intentional process of creating a new intervention program designed to address a specific educational need or target area (e.g., social skill development for adolescents with neurodivergence or foundational writing skills for early elementary-aged students). Development is appropriate when an important educational need is not adequately addressed by existing intervention programs or when available programs have meaningful limitations in effectiveness, feasibility, accessibility, equity, or contextual fit; a new program should therefore offer a clear and defensible contribution rather than merely duplicate existing approaches. Accordingly, high-quality intervention development begins with a deep understanding of the educational need being addressed and is grounded in relevant theory, empirical scholarship, and existing evidence regarding effective intervention strategies. The ultimate goal of development is to produce a comprehensive intervention program that has been iteratively refined and is ready for rigorous evaluation, practical implementation, and eventual dissemination if empirical evidence supports dissemination (Adelman & Taylor, 1994; Onken et al., 2014).

Developing an intervention program involves much more than creating instructional materials or assembling evidence-based strategies. Rather, it includes identifying and clearly defining the educational need, specifying the intended beneficiaries and desired outcomes, selecting and integrating evidence-based intervention strategies into a coherent program, designing and testing implementation procedures, creating implementation and training materials, engaging stakeholders throughout the design and development process, and ensuring that users will ultimately have reasonable access to all materials needed for high-fidelity implementation. In particular, this process merits attention to disparities in resources, language, accessibility, and cultural shifts, ensuring that the intervention is developed in a way that is accessible and meaningful to the target user group. When an intervention program reaches a mature stage of development, it should include, at minimum, a comprehensive intervention manual, specified training requirements and materials, implementation materials, the resources necessary for intended users to implement the program as designed, and guidance regarding appropriate adaptations when the program is used in contexts beyond those for which it was originally developed and evaluated (e.g., suggestions for age-appropriate adaptations of a program that was developed for use with elementary school-aged students but, if appropriately adapted, could potentially be beneficial for students in secondary school).

A defining characteristic of high-quality intervention development is the use of iterative design (Lyon & Koerner, 2016; Onken et al., 2014; Onken, 2022). After an initial prototype is created, the intervention is refined through multiple cycles of implementation, evaluation, stakeholder feedback, adaptive testing, and continuous improvement. These activities are essential to development because they provide the information needed to strengthen the intervention before formal evaluation studies of the sufficiently developed and stable program are conducted (Easterday et al., 2018). These activities also allow developers to integrate perspectives of professionals who may use the intervention and students aimed to benefit from it (e.g., students with lived experience)—and such information provides another way developers can consider the extent to which the intervention addresses issues of educational equity. Throughout this paper, we therefore view development as extending beyond creation of an initial prototype because iterative design is essential for moving the intervention to a sufficiently stable form that can then be subject to formal efficacy or effectiveness research (Philippakos et al., 2021). At that point, the intervention should be supported by well-developed implementation materials, have undergone substantial refinement through iterative processes, and be positioned for subsequent dissemination if evidence supports its effectiveness. Although an intervention program may continue to evolve after this point, later revisions should generally reflect substantive improvements—or possibly major adaptations for other populations or contexts—rather than ongoing refinement or minor tweaks of an otherwise unfinished program. If major revisions substantially alter the program, a revised intervention manual and a new edition of the program may also be warranted, along with additional evaluation of the revised program.

The amount of time required to move from an initial concept to a sufficiently developed and stable intervention program depends on many factors (e.g., the complexity of the intervention, available financial and personnel resources, opportunities for community engagement, and the amount of iterative testing required). In our experience, however, this process typically requires multiple years. Decisions made during development influence activities in every other domain of intervention work. Decisions regarding intervention targets, delivery format, implementation supports, required resources, and program documentation directly shape implementation procedures, evaluation designs, dissemination strategies, and ultimately whether intervention adopters will be able to implement the program successfully in real-world educational settings. Effective intervention development therefore requires anticipating the requirements of implementation, evaluation, and dissemination from the earliest stages of program design.

An applied example of the iterative cycle required to develop an intervention is drawn from Marraccini’s PrESR. Initial prototypes were based on cognitive behavioral therapy and exposure paradigms, and refined based on input from adolescents with lived experience, as well as school and hospital professionals (Marraccini et al., 2025). The prototype was then tested with adolescents and professionals during development (Marraccini et al., 2024a) and piloted with hospitalized adolescents (Marraccini et al., 2026). Feedback was again collected, leading to additional co-design activities meant to address the specific stressors faced by youth identifying as ethnic or racial minoritized and/or lesbian, gay, bisexual, transgender, queer or questioning (LGBTQ+).

Table 1 provides other illustrative examples of activities, scholarly areas, and cross-domain contributions associated with each of the four domains of intervention work. Together, these examples provide readers with an operational map illustrating the types of work that occur within each domain, the scholarly areas that inform that work, and the ways each domain supports the others throughout the intervention life cycle. As additional scholarly resources discussing intervention program development, user-centered design, and design-based research, readers may wish to consider Easterday et al. (2018), Lyon and Koerner (2016), McBride (2016), McKenney and Reeves (2019), Philippakos et al. (2021), or Razzouk and Shute (2012).

### 3.2. Intervention Program Implementation

Intervention program implementation refers to the process of delivering an intervention program within authentic educational settings. Although implementation is often viewed simply as the act of using an intervention program, it encompasses a much broader set of activities, including preparing implementers; providing training and coaching; monitoring implementation fidelity; documenting appropriate adaptations; identifying barriers and facilitators to implementation; and supporting sustained, high-quality program delivery. High-quality implementation extends beyond simply following prescribed procedures. It also involves attention to implementation quality, participant responsiveness, and the contextual factors that influence whether an intervention program can achieve its intended outcomes. Because intervention programs are intended for use in real-world educational settings, implementation is essential for determining whether a program can be delivered feasibly, consistently, and in ways that ultimately benefit the intended students.

Throughout this paper, it is helpful to distinguish between two broad phases of implementation: *implementation during development* and *post-development implementation*. Implementation during development occurs while an intervention program is undergoing iterative refinement. During this phase, implementation experiences provide essential information for improving program materials, implementation procedures, training supports, and other aspects of the intervention before formal evaluation of a sufficiently developed and stable program begins. Post-development implementation occurs after the intervention program has reached a sufficiently stable form for formal evaluation and, if supported by evidence, broader dissemination. During this phase, implementation focuses on understanding how the program can be delivered with fidelity across intended educational settings and student populations, what implementation supports are necessary (e.g., training, coaching, technical assistance), what adaptations are appropriate without compromising the intervention’s essential features, and what contextual factors influence successful implementation. Meaningful evaluation depends on implementation being sufficiently understood and documented, and knowledge gained through post-development implementation also provides the foundation for successful dissemination and scaling.

Both phases of implementation draw upon concepts and methods from implementation science, although their primary purposes differ. During intervention development, implementation science informs iterative refinement by identifying barriers, facilitators, stakeholder perspectives, and opportunities to improve the usability and feasibility of the intervention program. Following development, implementation science contributes to rigorous evaluation by documenting implementation fidelity, implementation quality, adaptations, and contextual influences on intervention outcomes while also generating the knowledge needed to support dissemination and scaling. Across both phases, implementation science allows for the development and testing of implementation strategies aimed at supporting uptake of the intervention. Together, these activities inform development while also supporting evaluation and dissemination throughout the intervention life cycle.

The Helping Early Literacy with Practice Strategies (HELPS) Program offers one example of a school-based reading intervention where research and development sought to thoroughly address the types of implementation considerations discussed throughout this subsection—and the teacher’s manuals offer relatively detailed implementation guidance for both the one-on-one and small-group HELPS programs. For a relatively condensed summary of this work, see Begeny et al. (2024b). As additional scholarly resources discussing intervention program implementation, readers may wish to consider Nilsen (2024) for a comprehensive book on implementation science, Landes et al. (2020) for an overview of implementation research designs, and Owens et al. (2014) for an overview of implementation science for school mental health.

### 3.3. Intervention Program Evaluation

Intervention program evaluation refers to the systematic collection and use of information to understand, improve, and ultimately determine the quality and effectiveness of an intervention program (Kidder et al., 2024). Although evaluation is often viewed primarily as determining whether an intervention “works,” evaluation serves a much broader purpose throughout the intervention life cycle. It informs decisions about program design, implementation, refinement, effectiveness, feasibility, equity, and dissemination. Like implementation, evaluation is not confined to a single point in the intervention life cycle but instead provides evidence throughout the intervention life cycle that supports decision-making across all four domains. Program evaluation occurs continuously during development to inform a final prototype and throughout implementation to evaluate the appropriateness of materials and procedures. High-quality evaluation therefore extends beyond measuring participant outcomes to also examining implementation quality, contextual influences, stakeholder perspectives, and the conditions under which an intervention program is most likely to produce meaningful and equitable benefits.

Similar to what we described of program implementation, it is useful to differentiate between two broad phases of evaluation: *evaluation during development* and *post-development evaluation*. Evaluation during development occurs while an intervention program is undergoing iterative refinement, with the primary goal of strengthening the program before it reaches a sufficiently stable form for formal efficacy or effectiveness research. During this phase, developers systematically gather information to improve the intervention’s overall effectiveness, enhance its usability and feasibility, strengthen implementation procedures and training supports, examine its usefulness across different student populations and educational contexts, and identify modifications that may improve future dissemination and scaling opportunities. Evaluation methods during this phase may range from informal observations and stakeholder feedback to structured pilot studies and methodologically rigorous quantitative, qualitative, or mixed-methods investigations. Some intervention programs may also benefit from rapid evaluation approaches, such as Rapid Iterative Testing and Evaluation (RITE), which can efficiently inform repeated cycles of refinement (Hanington & Martin, 2012; Huguet et al., 2023). Although rapid testing should not replace more comprehensive evaluation methods, it can represent a valuable component of high-quality iterative design.

Post-development evaluation begins after an intervention program has reached a sufficiently stable form for formal evaluation and broader implementation. During this phase, the primary purpose shifts from refining the intervention to determining whether it produces meaningful outcomes and understanding the conditions under which those outcomes can be achieved reliably, feasibly, equitably, and sustainably in authentic educational settings. Consequently, post-development evaluation draws heavily upon both intervention science and implementation science. Intervention science addresses questions regarding the efficacy, effectiveness, mechanisms, and intended impacts of the intervention program, whereas implementation science seeks to understand how the program can be delivered successfully across settings, populations, and delivery systems (Kidder et al., 2024). We encourage the use of quantitative, qualitative, and mixed-methods approaches during this phase (Kidder et al., 2024), but evaluation activities should generally employ formal and methodologically rigorous designs. This includes both high-quality program evaluation and formal research studies.

Although distinctions between program evaluation and research are not universally agreed upon—and often depend on context—we refer readers to Wanzer (2021) for a thoughtful discussion of these distinctions. For the purposes of this paper, we use *research* to refer to systematic investigations intended to contribute to the scholarly literature. *Program evaluation* refers to systematic evaluations that (a) draw upon established principles of high-quality evaluation (e.g., Alkin et al., 2024; Mertens et al., 2025) and (b) are intended to understand program impacts and other aspects of program quality in order to inform decision-making about the intervention program itself. Both forms of evaluation provide essential evidence regarding an intervention program’s quality, effectiveness, implementation, and potential for broader dissemination. Although research and program evaluation often employ similar methodological approaches, they typically differ in their primary purpose. Research is generally intended to produce knowledge for a scholarly community in ways that advance existing scholarship, whereas program evaluation is often intended to inform decisions about a particular intervention program or its implementation. Within the broader context of intervention work, research and program evaluation therefore serve complementary purposes, together providing the evidence needed to strengthen intervention programs, advance intervention science, and support informed decision-making by intervention adopters.

For intervention adopters, evaluation should not end once an intervention program has been selected and implemented. Rather, continued program evaluation within the local educational context represents both a practical and ethical responsibility because intervention effectiveness may vary across settings, student populations, implementers, and other contextual factors—all of which have implications for educational equity. Evaluating interventions with varied populations, interventionists, settings, implementation conditions, and research teams is critical to inform broader conclusions about the effectiveness of an intervention. Consequently, high-quality intervention programs should provide adopters with feasible guidance for monitoring implementation and evaluating local program outcomes, thereby allowing continued learning that can inform local decision-making while potentially even contributing to the broader knowledge base surrounding the intervention program.

Evaluation findings frequently identify opportunities for continued program refinement, improve implementation guidance and training, strengthen dissemination planning, and inform decisions made by intervention adopters regarding whether an intervention program is appropriate for their particular educational context. Meaningful evaluation therefore requires careful attention to how an intervention program was developed and implemented, while simultaneously providing the evidence needed to strengthen future intervention work across the intervention life cycle. Examples of activities, scholarly areas, and cross-domain contributions associated with intervention program evaluation are shown in Table 1. As additional scholarly resources discussing intervention program evaluation, readers may wish to consider the CDC’s program evaluation framework (Kidder et al., 2024) or comprehensive books covering both theory and application of program evaluation (e.g., Alkin et al., 2024; Mertens et al., 2025).

### 3.4. Intervention Program Dissemination

Intervention program dissemination refers to the intentional processes used to increase awareness, access, understanding, adoption, and sustained use of an intervention program—or information about that program—among specific target audiences (Purtle et al., 2022). Although dissemination is often viewed as the final stage of intervention work, it should instead be understood as a domain that is interconnected with development, implementation, and evaluation throughout the intervention life cycle. For example, dissemination activities commonly occur during intervention development through presentations to stakeholders, conference presentations describing early findings, or sharing prototype materials to obtain feedback. Likewise, implementation and evaluation findings are often disseminated as they emerge to inform practitioners, researchers, and other interested audiences. Nevertheless, dissemination becomes especially important once an intervention program has reached a sufficiently mature stage of development (e.g., a fully manualized program) and has accumulated at least some evidence of efficacy or effectiveness. At that point, dissemination serves as the primary avenue through which the products and knowledge generated during development, implementation, and evaluation become available to broader audiences to ensure effective and equitable adoption of evidence-based interventions (Kwan et al., 2022). Without effective dissemination, even well-developed and rigorously evaluated intervention programs are unlikely to influence educational practice or improve student outcomes at scale.

Compared to the other three domains, dissemination remains relatively underdeveloped as an area of scholarship, particularly within education. Although dissemination and implementation (D&I) science has grown substantially over the past two decades—especially within public health and medicine (Purtle et al., 2022)—research specifically focused on dissemination remains conceptually fragmented. In their recent scoping review of empirical dissemination studies using quantitative or mixed methods designs, Baumann and colleagues (2022) reported that only 89 studies met their inclusion criteria, with fewer than half using an explicit dissemination framework, and multiple definitions and conceptual frameworks were represented across the literature. Similar observations have been noted elsewhere (e.g., Scott et al., 2024; Tabak et al., 2012), highlighting the continued need for clearer terminology and more unified conceptualizations of dissemination. Rather than attempting to reconcile competing frameworks or definitions that currently exist within the literature, we intentionally adopt a practical conceptualization of dissemination that aims to emphasize clarity and usefulness for school-based intervention scientists and adopters.

A distinguishing feature of dissemination, compared to the other three domains, is that effective dissemination is fundamentally audience-dependent. The type of information communicated, the methods used to communicate it, and the desired outcomes of dissemination differ substantially depending on whether the intended audience consists of researchers, educators, school leaders, policymakers, families, or members of the broader public. For example, prioritizing the needs and perspectives of varying stakeholders at the onset and throughout the dissemination process helps anticipate and address dissemination challenges related to educational equality or equity (Kwan et al., 2022).

We propose four broad categories of dissemination that collectively encompass the primary dissemination audiences relevant to school-based intervention programs. *Scholarly dissemination* aims to advance intervention and implementation science by communicating findings through peer-reviewed publications, conference presentations, technical reports, scholarly books, and related scientific outlets. *Practice dissemination* focuses on current and potential intervention users by providing manuals, training opportunities, implementation guidance, technical assistance, websites, webinars, and other resources that facilitate informed adoption and high-quality implementation. *Public dissemination* seeks to increase awareness and understanding of intervention programs among families, community organizations, media outlets, and the broader public through accessible forms of communication. Finally, *policy dissemination* targets decision-makers such as school boards, educational agencies, governmental organizations, and funding bodies by communicating evidence that may influence policy decisions, funding opportunities, funding priorities, and broader systems-level adoption. Although these categories are not mutually exclusive, they provide a practical framework for conceptualizing the primary pathways through which intervention programs and the knowledge generated about them are disseminated. Successful dissemination is not defined simply by the amount of information shared, but by whether dissemination efforts effectively reach their intended audiences and support appropriate awareness, understanding, adoption, implementation, or decision-making.

Early dissemination activities (e.g., sharing intervention concepts or early discoveries with school-based community partners) frequently generate stakeholder feedback that strengthens intervention development. Likewise, dissemination of implementation findings can improve implementation guidance, training resources, and future implementation efforts. Once a manualized intervention program has accumulated evidence of efficacy or effectiveness, dissemination of evaluation findings becomes essential for informing decisions regarding adoption, funding priorities, policy, and future research. Ultimately, intervention program dissemination is not simply “getting the word out.” Rather, it represents the means through which the knowledge and products generated during development, implementation, and evaluation extend beyond the original program developers to produce broader scientific knowledge, educational improvement, and societal impact.

As a specific example of one school-based intervention framework, Positive Behavioral Interventions and Supports (PBIS) illustrates how an approach can move from development and empirical study to widespread implementation and scale-up, while also highlighting the continuing importance of implementation supports, fidelity, infrastructure, and evaluation after dissemination (Pas et al., 2019; Sugai & Horner, 2020). Other scholars also provide useful guidance, discussions, and empirical contributions pertaining to the dissemination of interventions (e.g., Baumann et al., 2022; Cassar et al., 2024; Purtle et al., 2022; Richmond & Steffen, 2020; Sam et al., 2020).

### 3.5. Synthesizing the Four Domains of Intervention Work

**Figure 1:**
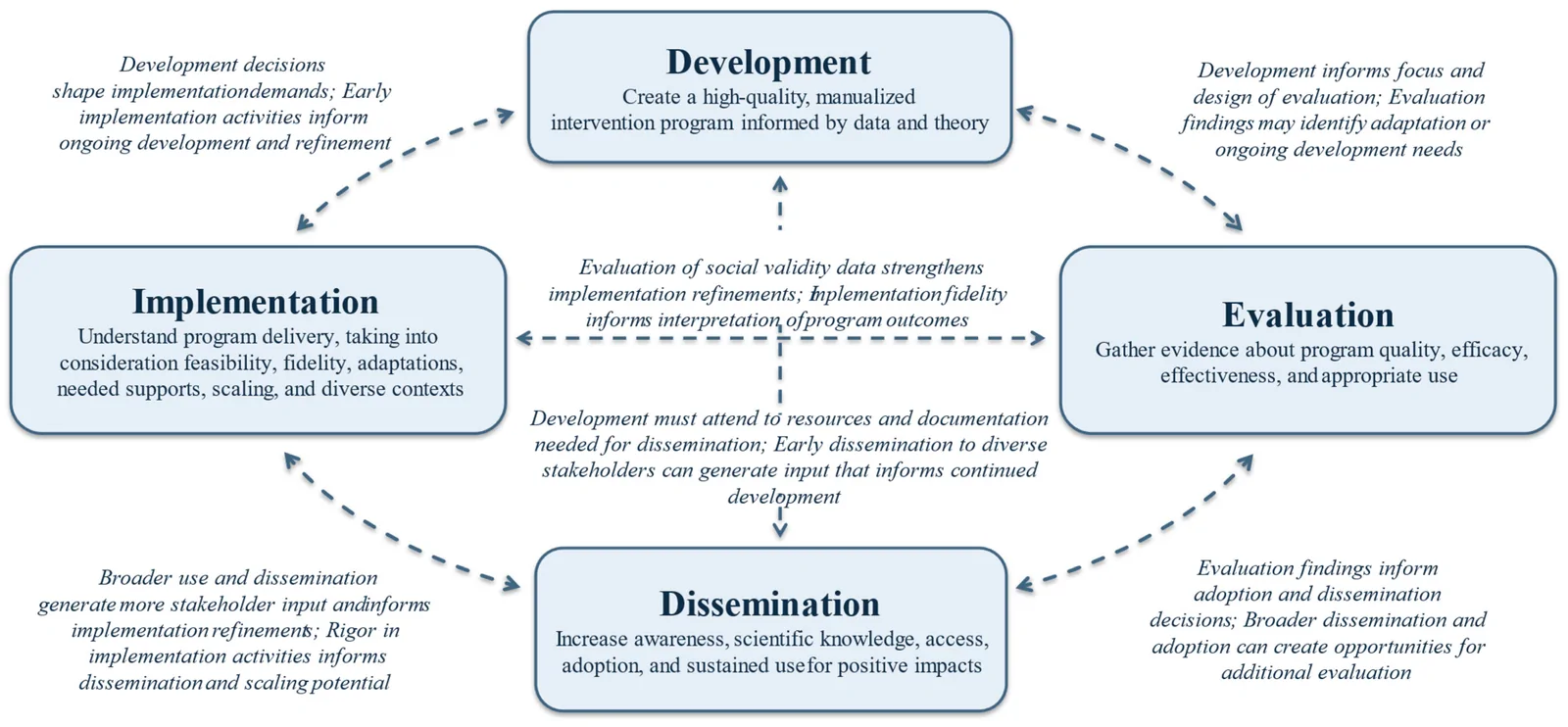
Examples of Cross-Domain Relevance During Intervention Work.

***Note***. Each box identifies a primary domain of intervention work and summarizes its primary goal (see Table 2). Dashed bidirectional arrows indicate cross-domain relevance and interconnectedness; italicized text provides selected illustrative examples of how work within each domain can inform work within another. See Table 1 and the accompanying text for additional examples.

Taken together, the four domains described above provide a functional conceptualization of intervention work that emphasizes not only what occurs throughout the intervention life cycle, but also how the domains collectively contribute to the production, implementation, evaluation, dissemination, and adoption of high-quality intervention programs. Rather than viewing the four primary domains as isolated or strictly sequential activities, we encourage readers to conceptualize them as complementary and highly interconnected domains. Decisions made within one domain shape activities, opportunities, and challenges within the others, and high-quality intervention work therefore requires attention to all four domains. Although intervention scientists and intervention adopters will often engage more directly with some domains than others, understanding the purpose, products, and interconnections of all four domains provides a stronger foundation for intervention science, implementation science, and evidence-based educational practice. Figure 1 provides examples of cross-domain relevance during intervention work.

Equally important, each domain produces a distinct but complementary form of knowledge that supports subsequent intervention work. Development produces a theory- and data-informed intervention program that is sufficiently mature for broader implementation and evaluation. Implementation generates practical knowledge regarding how the program can be delivered with fidelity, feasibility, and appropriate adaptations in real-world educational settings. Evaluation provides evidence regarding the intervention program’s quality, effectiveness, implementation, and appropriate use, thereby informing continued refinement during development as well as adoption decisions once the intervention program has reached a sufficiently mature stage. Dissemination extends the knowledge and products generated within the other three domains to broader scientific, educational, policy, and public audiences, thereby increasing opportunities for adoption, sustained implementation, and societal impact. Collectively, these complementary forms of knowledge strengthen intervention science, improve educational decision-making, and provide intervention scientists and intervention adopters with a shared conceptual understanding of high-quality intervention work.

Table 2 further synthesizes these four domains by summarizing their primary goals, principal products, and key areas of relevance for intervention scientists and adopters. Whereas Table 1 provides an operational map of intervention work, Table 2 provides a conceptual map by illustrating what each domain is intended to accomplish, what it produces, and why it matters. Next, we summarize some important practical questions and considerations connected to each domain, and we highlight why these questions matter for both intervention scientists and adopters.

## 4. Key Questions and Considerations for Intervention Scientists and Intervention Adopters

The questions and considerations presented throughout the following sections are intended to translate the conceptual perspective developed throughout this paper into opportunities for practical reflection. The first author developed these questions from recurring considerations that arose through his work in intervention development, implementation, evaluation, and dissemination—and subsequently refined them through engagement with scholarship relevant to these domains. Rather than seeking to establish an exhaustive set of activity-focused questions or a formally derived consensus, a smaller set of questions was intentionally distilled to reflect broader principles underlying high-quality intervention work. Collectively, these questions encourage readers to consider not only what should be accomplished within each domain, but also how work conducted within one domain can intentionally support and strengthen work occurring throughout the others.

Important to highlight, these questions are not intended to prescribe a single course of action or function as a checklist of tasks to be completed. Instead, they are designed to support thoughtful professional judgment by encouraging readers to integrate available evidence, contextual realities, professional expertise, and stakeholder perspectives when making decisions about intervention work. Many of the questions do not have universally correct answers, nor will every question be equally relevant to every intervention program or educational setting. Rather, the value of these questions lies in encouraging intentional planning, critical reflection, and informed decision-making throughout intervention work.

High-quality intervention work often involves iterative cycles of planning, implementation, evaluation, reflection, and refinement as intervention programs mature and new knowledge emerges. New evidence, implementation experiences, stakeholder perspectives, local evaluation findings, or changing educational contexts may appropriately prompt intervention scientists and intervention adopters to revisit earlier decisions. From our perspective, revisiting earlier decisions should not be viewed as evidence that previous efforts were inadequate, but as an expected and often valuable response when new evidence or contextual circumstances warrant reconsideration. At the same time, intervention programs that consistently demonstrate strong evidence of effectiveness, feasibility, and the overall social validity required for sustained implementation may need little to no refinement across many years, beyond ongoing monitoring and periodic evaluation that should occur with any program over time.

To further support practical reflection and professional judgment, our paper concludes with separate Reflection Guides for intervention scientists and intervention adopters. These guides synthesize the conceptual and practical ideas presented throughout the paper into resources that can support planning, informed decision-making, ongoing self-assessment, and thoughtful reflection. Although tailored to the unique roles of these two audiences, both guides are grounded in the same conceptual perspective presented throughout this paper and are intended to promote a shared understanding of high-quality intervention work.

### 4.1. Intervention Program Development: Key Questions and Considerations

#### 4.1.1. What Educational Need Is the Intervention Intended to Address, and What Existing Knowledge Supports Its Purpose and Design?

Intervention development should begin with a clearly articulated educational need and a compelling rationale for why that need warrants attention. Before creating a new intervention program, developers should examine the strongest available knowledge concerning related programs, strategies, theories, and educational practices, including relevant systematic reviews, meta-analyses, individual studies, and research conducted across varied cultural and international contexts. This process can identify promising intervention components, limitations of existing approaches, populations or settings that have been insufficiently served, and questions that remain unanswered. Existing intervention-development models or frameworks may help organize this work, but they cannot replace the professional and context-specific judgment required to determine what knowledge is most relevant, whether a genuinely new program is needed, or how urgently development should proceed. Clearly documenting the need and intellectual foundation of the intervention program also supports the other domains. For instance, it establishes the rationale for subsequent research questions within intervention and implementation science, clarifies the outcomes against which the intervention should be evaluated, and helps prospective adopters determine whether the program addresses an important need within their own educational settings.

#### 4.1.2. How Has the Intervention Been Collaboratively Designed for the People, Settings, and Systems in Which It Will Be Used?

Intervention programs are more likely to be usable, feasible, equitable, and educationally meaningful when their development reflects sustained attention to the people who will deliver and receive them, as well as the settings and systems in which implementation will occur. Depending on the intervention and stage of development, this may involve community-engaged scholarship, research–practice partnerships, user-centered design, participatory design, or other forms of collaboration with educators, students, families, administrators, and relevant community partners. Meaningful collaboration extends well beyond requesting feedback after major decisions have already been made; rather, it creates opportunities for stakeholder knowledge and experience—and, in some cases, co-development—to shape intervention goals, procedures, materials, training supports, and implementation expectations. Development should also account for authentic educational conditions, including staffing, schedules, available resources, technology, existing systems of support, and the cultural, linguistic, and experiential diversity of intended recipients and users. Attention to these factors does not ensure that an intervention will fit every setting, but it increases the likelihood that the resulting program can be implemented responsibly and provides prospective adopters with clearer information for judging its relevance, feasibility, and fit within their local contexts.

#### 4.1.3. What Must Be Sufficiently Developed, Tested, and Documented Before the Intervention Is Ready for Consistent Implementation, Rigorous Evaluation, and Responsible Dissemination?

Before an intervention is implemented consistently, evaluated rigorously, or disseminated broadly, its essential procedures, materials, and implementation supports should be developed and tested to a degree appropriate for those purposes. At minimum, program users should be able to identify the program’s core components, the procedures necessary for implementation, the elements that may be adapted, the materials required by interventionists and recipients, and the conditions under which the program is expected to be feasible and useful. Manuals and related resources should provide practical guidance concerning training, coaching, implementation fidelity, permissible adaptations, common implementation challenges, and local evaluation. Pilot testing and early implementation experiences can help determine whether these resources are sufficiently clear, complete, and usable before stronger claims about effectiveness or readiness for broader adoption are made. Sufficient development does not mean that an intervention must thereafter remain unchanged; subsequent evidence and experience may appropriately warrant refinement. However, frequent changes to essential components may indicate that additional development and pilot testing are needed, and substantial revisions should be documented transparently—potentially as a new program edition. Establishing an appropriately stable intervention helps protect the interpretability of evaluation findings, supports responsible dissemination, and gives adopters a sufficiently consistent program to implement and assess.

### 4.2. Intervention Program Implementation: Key Questions and Considerations

#### 4.2.1. How Will Implementation Practices Promote Usability, Feasibility, Social Validity, and Equitable Use in Authentic Educational Settings?

Implementation provides opportunities to examine how an intervention functions when used by intended interventionists and recipients under authentic educational conditions. Knowledge from implementation science and iterative design can help intervention scientists and adopters anticipate barriers, study implementation processes, and respond thoughtfully to challenges without requiring expertise in every area of this expansive scholarship. Relevant information may be gathered through implementation data, local evaluation, observations, interviews, surveys, and ongoing consultation with interventionists, recipients, families, administrators, and community partners. Collectively, these sources can clarify whether the intervention is usable, feasible, acceptable, appropriate, and socially valid—and whether factors such as staffing, schedules, technology, organizational systems, available resources, cultural responsiveness, or accessibility create unequal opportunities to implement or benefit from the program. Early and ongoing implementation experiences may identify practical improvements to intervention procedures or supports, but they may also affirm that the existing program functions well under the conditions examined. In either case, systematically learning from implementation strengthens decisions about continued use, subsequent evaluation, responsible dissemination, and the settings or populations for which claims about the intervention are warranted.

#### 4.2.2. What Training, Coaching, and Ongoing Supports Are Needed for Intended Interventionists to Implement the Program with Appropriate Quality and Fidelity?

Successful implementation depends partly on clearly identifying who is expected to deliver the intervention and determining the knowledge, skills, preparation, and ongoing support those individuals need. Training may include manuals, workshops, online modules, demonstrations, active practice, feedback, and other learning activities; continued support may involve coaching, consultation, booster sessions, professional learning communities, or collaborative problem-solving. The appropriate combination depends on the intervention, intended interventionists, implementation context, and mechanisms through which the supports are expected to improve knowledge, motivation, skill, and practice (Sims et al., 2021). Because one-time training is often insufficient for developing and sustaining high-quality implementation, intervention scientists and adopters should consider how interventionists will receive feedback and assistance as challenges arise (Langreo, 2022). At the same time, training and coaching expectations must remain feasible within the time, staffing, and resource constraints of authentic educational settings. Clearly documenting the content, duration, sequence, intended outcomes, and resource requirements of these supports enables their effectiveness to be evaluated, helps prospective adopters judge whether they can implement the program responsibly, and increases the likelihood that implementation can be replicated and sustained across varied users and settings.

#### 4.2.3. How Will Implementation Experiences and Data Guide Adaptation, Refine Fidelity Expectations, and Strengthen Subsequent Intervention Work?

High-quality implementation requires sufficient consistency to preserve essential functions of the intervention while allowing appropriate responsiveness to differences among interventionists, recipients, and educational contexts. Accordingly, intervention programs should clearly identify their core components; specify which elements may be adapted; and define implementation fidelity through dimensions relevant to the program, such as adherence, dosage, quality of delivery, participant responsiveness, and differentiation from other practices. Determinations about which components are considered core should be grounded in the intervention’s theoretical or conceptual rationale; hypothesized mechanisms of action; and, when available, empirical evidence linking particular components to meaningful outcomes. As evidence accumulates, implementation and evaluation findings may further clarify which components are essential for preserving the intervention’s intended functions and which can be adapted without undermining effectiveness. Implementation data can then help determine whether these expectations are both practically feasible and empirically defensible.^4^ When difficulties or disappointing outcomes occur, professional judgment is needed to consider whether they reflect (a) inadequate implementation, (b) insufficient training or coaching, (c) an inappropriate local adaptation, (d) a mismatch between the intervention and its context of use, or (e) a limitation of the program itself. When adaptations appear successful, they may warrant further study before being incorporated into broader program guidance. Ultimately, knowledge generated through systematic and rigorous examination of implementation can refine fidelity standards, intervention materials, the program manual, training supports, evaluation designs, and dissemination guidance. Thus, implementation should not be viewed merely as carrying out a previously developed program; it is also a major source of knowledge for determining what should remain stable, what may appropriately change, and how subsequent intervention work can be strengthened.

### 4.3. Intervention Program Evaluation: Key Questions and Considerations

#### 4.3.1. What Should Evaluation Reveal About the Intervention’s Effects; How They Are Produced; And Whether They Vary Across People, Settings, and Implementation Conditions?

Evaluation should be guided by questions that align with the educational need, goals, theoretical foundation, and intended mechanisms of the intervention program. Although formative and early-stage studies may appropriately emphasize feasibility, acceptability, appropriateness, social validity, and preliminary outcomes, evaluation should ultimately determine whether the intervention produces meaningful educational effects. Information about implementation is also essential because intervention outcomes cannot be interpreted fully without understanding how, by whom, and under what conditions the program was delivered, including whether implementation with adequate fidelity was feasible across those conditions. As the evidence base develops, evaluation may examine mechanisms through which effects occur; potential mediators and moderators; unintended outcomes; and whether findings vary across recipients, interventionists, settings, or implementation conditions. However, these secondary questions should be pursued when the study’s purpose, design, and statistical power can support them, rather than at the expense of adequately examining the intervention’s primary effects. Input from educators, students, families, community partners, and other stakeholders can help identify outcomes and contextual factors that are both scientifically meaningful and educationally relevant (Kidder et al., 2024). Evaluation should also examine whether historically underserved populations are adequately represented and whether access, implementation, or outcomes differ in ways that may advance or undermine educational equity.

#### 4.3.2. What Combination of Methods, Studies, and Replications Is Needed to Support Credible Conclusions Without Extending Claims Beyond the Available Evidence?

No single study or research method can answer every important question about an intervention program. Even a substantial body of well-conducted studies is unlikely to address every relevant population, context, outcome, or implementation condition, and additional research may therefore extend or clarify what is known about the intervention. Quantitative, qualitative, and mixed methods can generate complementary knowledge, and the most appropriate methods and designs will depend on the questions being addressed and the maturity of the intervention and its evidence base. Formative studies and pilot evaluations may initially examine feasibility, implementation, measurement, and preliminary outcomes. When findings from these early phases of research demonstrate sufficient promise, increasingly rigorous efficacy and effectiveness studies can test intervention effects across more authentic and varied conditions. These forms of research are better understood as existing along a continuum than as perfectly distinct categories. Credible conclusions also require systematic replication across populations, interventionists, cohorts, educational settings, implementation conditions, and research teams, including teams with little or no involvement from the intervention developers. Collectively, the strength, consistency, relevance, and limitations of the available studies should determine what may responsibly be claimed about intervention effectiveness, generalizability, and evidence-based status. The body of evidence supporting an intervention may also evolve as new research and high-quality program evaluations become available, requiring conclusions to reflect the strongest and most complete evidence available at a given time. Clearinghouse classifications and similar summaries may inform judgments about intervention effects, but they should not replace critical examination of the broader body of underlying research or justify conclusions that extend beyond the outcomes, populations, settings, and conditions that have actually been studied.

#### 4.3.3. How Will Evaluation Extend Beyond Initial Developer-Led Research to Support Local Decision-Making, Continued Learning, and Responsible Dissemination?

Evaluation should not end when intervention developers complete their initial research studies or begin disseminating the program to interested schools. Intervention programs and their manuals should provide adopters with feasible guidance, measures, and decision-making resources for examining both implementation and meaningful outcomes within authentic educational settings. Local evaluation can help educators determine whether the intervention is addressing identified needs, being implemented adequately, producing equitable and educationally meaningful benefits, and remaining feasible within available resources and organizational conditions. When interpreted alongside the broader research evidence, local findings can inform professional judgments about maintaining, expanding, adapting, reducing, or discontinuing use of the program. Research–practice partnerships and other forms of sustained collaboration can strengthen this work by ensuring that evaluation questions address authentic problems of practice and that the benefits and demands of research are considered and distributed responsibly across researchers and those involved with the research, such as school partners or intervention recipients (Coburn & Penuel, 2016). Dissemination practices can also facilitate continued evaluation by making intervention materials, research findings, implementation guidance, and opportunities for independent investigation sufficiently accessible. Continued evaluation does not presume that every well-supported intervention requires ongoing modification; local monitoring and periodic assessment may instead affirm that the program is functioning effectively and should remain stable. In this way, evaluation supports both responsible adoption decisions and continued learning about the circumstances in which an intervention can be used effectively and sustained over time.

### 4.4. Intervention Program Dissemination: Key Questions and Considerations

#### 4.4.1. When Is the Intervention Program Ready for Broader School-Based Use, and What Information and Resources Must Accompany Its Dissemination into Practice?

An intervention program may be implemented in several schools during development, implementation research, and initial evaluation before it is ready for broader use with substantially less direct involvement from its developers. Determining readiness for this transition requires professional judgment about whether work across the other domains has produced a sufficiently developed and stable program, feasible implementation procedures, and credible evidence to support responsible use in a broader set of educational settings. As discussed earlier in this paper, dissemination can serve several distinct purposes; however, the considerations addressed here pertain primarily to *practice dissemination* (i.e., the processes through which current and prospective intervention users receive the information, materials, training opportunities, implementation guidance, technical assistance, and related resources needed for informed adoption and high-quality implementation). These resources should communicate the intervention’s purpose; evidence; limitations; intended users and recipients; implementation requirements; fidelity expectations; permissible adaptations; and the populations, settings, and conditions in which it has been studied. The program manual and associated materials should also support local evaluation and clarify whether the intervention is appropriate for standard-protocol use or requires additional assessment, expertise, or individualized decision-making. Importantly, readiness for carefully supported practice dissemination does not necessarily indicate readiness for widespread scaling; broader use can remain purposeful and limited while implementation and evaluation continue to generate knowledge that informs subsequent dissemination decisions.

#### 4.4.2. How Will Dissemination Strategies Promote Equitable Access, Evidence-Driven Adoption, and Responsible Use of the Intervention?

Dissemination strategies influence not only whether potential users learn about an intervention, but also who can access it, what information they receive, and whether they have realistic opportunities to implement it successfully. Intervention scientists should therefore consider which communication channels, organizations, and dissemination models are most appropriate for the program and its intended users. Possibilities may include university-supported dissemination, nonprofit organizations, commercial partnerships, open-access resources, or blended approaches, each of which carries different implications for program availability, quality assurance, sustainability, and cost. Copyright and licensing arrangements may help protect the integrity of intervention materials, but costs associated with materials, technology, training, coaching, or technical assistance can also create barriers—particularly for schools and communities with fewer resources. Equitable dissemination consequently requires attention not only to whether the program is equally available, but also to whether intended adopters can meaningfully access, implement, and benefit from it. Responsible dissemination materials should accurately communicate the strength and limitations of the evidence rather than rely on exaggerated promotional claims, selective presentation of favorable findings, misleading information, or misinformation. Likewise, adopters should critically examine whether decisions are based primarily on evidence of program value and local fit rather than popularity, persuasive marketing, institutional habit, political pressure, prior financial investment, or other influences unrelated to intervention quality and outcomes.

#### 4.4.3. How Will Dissemination Support Continued Evaluation, Strategic Scaling, and Responsible Stewardship of the Intervention over Time?

Dissemination can extend the reach of an intervention while also creating new opportunities to examine its implementation and outcomes across varied populations, interventionists, and educational settings. Making program materials, implementation guidance, research findings, and evaluation resources sufficiently accessible may support high-quality local evaluation, systematic replication, and independent research conducted by teams with little or no involvement from the intervention developers. Knowledge generated through broader use can help clarify the conditions under which the program is effective, feasible, and sustainable and can inform subsequent decisions about implementation supports, evaluation priorities, dissemination guidance, and potential scaling. Strategic scaling should occur only when the available evidence and implementation infrastructure are sufficient to support expansion without compromising program quality or equitable access. This requires thoughtful selection of organizational partners, funding arrangements, training systems, technical-assistance structures, and processes for maintaining fidelity and quality across settings. Responsible stewardship also includes establishing who will oversee program updates, version control, communication of new evidence, user support, and decisions concerning future revisions. Continued learning may sometimes warrant refined guidance, additional resources, or a new program edition, but broader use may also affirm that a well-supported intervention should remain substantially stable. Thus, dissemination is not simply the distribution or promotion of an intervention; it is an ongoing responsibility to preserve program integrity, support informed and effective use, and ensure that knowledge generated through dissemination continues to strengthen intervention work.

### 4.5. Using the Reflection Guides to Extend the 12 Key Questions and Considerations

The Reflection Guides for intervention scientists (Appendix A) and intervention adopters (Appendix B) extend our 12 key questions and considerations by translating the functional conceptualization presented in this paper into practical prompts for planning, informed decision-making, collaborative discussion, and periodic self-assessment. Although organized around the same four domains of intervention work, the guides are tailored to the distinct roles and responsibilities of scientists and adopters, and they are intended to support professional judgment rather than function as exhaustive checklists. When adopters cannot answer a question using the program manual, publicly available evidence, or their knowledge of the local context, we suggest they should seek additional information by contacting the intervention developer(s) or those responsible for practice dissemination of the respective program. Providing clear and transparent responses—or acknowledging when relevant knowledge or evidence is not yet available—should be viewed as an important responsibility of intervention developers and disseminators.

For intervention scientists and adopters working under substantial resource constraints, the Reflection Guides should not be interpreted as requiring every consideration to be addressed simultaneously or with equal depth. Priorities should instead be guided by the intervention’s stage of development, intended use, local context, available resources, and the likely consequences of particular decisions. Greatest attention should be given to the considerations most essential for intervention quality, feasible and equitable implementation, credible evaluation, and responsible dissemination. Rather than treating all items in the Reflection Guides as expectations that must be fulfilled at once, more resource-intensive activities may sometimes need to be sequenced over time, pursued through partnerships or additional collaboration, or identified transparently as areas requiring future attention. In the next section, we address some of these same issues in the context of community-engaged intervention research.

## 5. Calls to Action and Concluding Comments

Far too many students throughout the world are not realizing their educational potential, and substantially more must be done to support them (National Center for Education Statistics, 2024; OECD, 2023, 2024; ProLiteracy, 2025). Although these unmet educational needs extend across student populations, persistent disparities demonstrate that race, family socioeconomic status, and other conditions continue to contribute to the educational marginalization of many students and communities (Klees & Qargha, 2014; Ladson-Billings, 2006; OECD, 2023; Suzuki & Begeny, 2025). Most educators work tirelessly to provide the best possible education to the students they serve, but their efforts must be supported by greater access to high-quality intervention programs that are grounded in rigorous development, implementation, evaluation, and dissemination. This need is not new. Reflecting on the substantial attention education research has devoted to students from historically marginalized communities, Ladson-Billings observed, “We seem to study them but rarely provide the kind of remedies that help them to solve their problems” (Ladson-Billings, 2006, p. 3). Two decades later, that general assessment and its accompanying call to action remain strikingly relevant. Researchers and practitioners in education and psychology have long noted that substantially more scholarship is needed to examine intervention and prevention programs in schools (Begeny et al., 2018; Bliss et al., 2008; Strein et al., 2003; Villarreal et al., 2013).

As one very current example, although the Special Issue in which this paper is published—*Promoting Social-Emotional and Academic Support in Educational Settings*—offers meaningful scholarship and important insights, it also illustrates a broader imbalance: at the time of writing, only one of the 17 articles included in the Special Issue directly examined the effects of an intervention program. Responding to this imbalance will require more individuals—including university researchers and others positioned to lead such efforts—to pursue the sustained work of developing high-quality intervention programs. Intervention work is undeniably complex, multidisciplinary, and resource intensive, but its complexity should not dissuade researchers or emerging program developers. Rather, it underscores the need for thoughtful preparation, community partnerships, multidisciplinary collaboration, professional judgment, and continued learning across the four domains described in this paper. Universities also have an important role in supporting this work. Community-engaged intervention research often requires sustained partnerships, iterative development and evaluation, replication studies, as well as other activities that can require substantial time and may not align easily with traditional institutional reward structures and expectations for scholarly productivity (Jameson et al., 2010; Wang et al., 2026; Wendling, 2023; Worthen et al., 2025). Institutional priorities and reward structures that better recognize the rigor, time, and public value of this work could therefore strengthen intervention science while also advancing the public purposes of higher education (Bell & Lewis, 2023; Boyer, 1996; Kirchherr, 2018).

Throughout this work, contemporary evidence and dialogue concerning educational equity must remain at the forefront. Intervention scientists should continually ask which students are being systematically denied opportunities to realize their learning potential; if and how intervention programs can address those inequities; and whether decisions across development, implementation, evaluation, and dissemination expand meaningful opportunities for students who have been educationally marginalized. These considerations are particularly important in schools and communities facing substantial resource constraints or structural barriers to educational and health-related supports. Such disparities can affect not only access to intervention programs, but also the resources and organizational supports available for high-quality implementation, evaluation, adaptation, and sustained use (Thayer et al., 2022; Sridhar et al., 2025). Attention to equity therefore requires considering these realities throughout the intervention life cycle rather than treating equity as a consideration separate from intervention work.

Intervention adopters likewise need support when navigating complex program-selection decisions, but they also have a professional responsibility to examine those programs carefully. Informed and equitable adoption requires attention to credible evidence, local needs, and contextual fit, as well as reasonable access to the information and resources needed to assess intervention quality. Schools and communities remain vulnerable when organizations with substantial resources and incentives for marketing and sales disseminate programs whose promotional reach exceeds the rigor of the programs’ development or supporting evidence. The need, therefore, is not merely for more intervention programs, but for more programs worthy of the trust that educators, students, families, and communities place in them—and for informed adopters who can distinguish those programs from less defensible alternatives. We hope the functional conceptualization, key questions, and Reflection Guides offered in this paper provide one additional resource for navigating both the rewards and complexities of intervention work. Students, educators, families, and communities depend on scientists and adopters fulfilling these shared responsibilities if educational opportunities are to become more equitable and effective.

## Figures and Tables

**Table 1:** Illustrative Activities, Scholarship, and Cross-Domain Contributions Across the Four Domains of Intervention Work.

Domain	Examples of Key Activities	Examples of Important Areas of Scholarship ^a^	How Activities Connect to Intervention Work within the Other Domains
**Development**	Develop an intervention prototype. Refine the program through rigorous iterative design processes. Establish sustainable community partnerships that inform development. Develop implementation materials, training resources, implementation supports, and evaluation guidance. Produce a comprehensive intervention manual. Finalize a mature intervention program ready for formal evaluation.	Theoretical and empirical scholarship relevant to the target area Human-centered design and/or design-based research Iterative design methodologies Community engagement and partnerships ^b^ Scholarship related to equity, diversity, inclusion, and accessibility Relevant educational policy (when applicable)	Anticipates implementation by developing manuals, implementation supports, training materials, fidelity guidance, and adaptation guidance. Produces an intervention program ready for high-quality implementation and rigorous evaluation. Creates the resources and documentation necessary for dissemination and broader adoption.
**Implementation**	Implement the intervention during iterative development and after program finalization. Monitor implementation fidelity across different interventionists and educational contexts. Evaluate implementation feasibility, usability, acceptability, and appropriateness. Develop, refine, and evaluate training and coaching supports. Document appropriate adaptations across educational contexts. Identify implementation barriers and facilitators.	Implementation science School-based systems and organizational change Project management Professional learning and adult education Fundraising and sustainability planning	Generates knowledge that strengthens ongoing intervention development and refinement. Provides essential information needed for rigorous evaluation. Identifies implementation supports, adaptations, and training resources that facilitate dissemination, adoption, and scaling.
**Evaluation**	Collect implementation and outcome data throughout the intervention life cycle. Evaluate intervention quality during iterative development. Conduct formal efficacy and effectiveness studies. Conduct high-quality program evaluation within authentic educational settings. Examine implementation quality, contextual influences, and education equity. Provide guidance for ongoing, feasible local program evaluation by intervention adopters.	Intervention science Research design (experimental, quasi-experimental, single-case, mixed methods) Qualitative research methodologies. Program evaluation Measurement and psychometrics Survey methodology and statistics	Provides evidence that informs continued intervention refinement. Strengthens implementation through improved understanding of fidelity, feasibility, adaptations, and context. Produces evidence necessary for dissemination, adoption, funding, and policy decisions.
**Dissemination**	Disseminate findings to scholarly audiences. Develop manuals, websites, webinars, and implementation resources. Provide professional development and technical assistance. Support intervention adoption, strategic scaling, and long-term sustainability. Communicate findings to policymakers, funding organizations, and educational leaders. Engage families, community partners, and the broader public.	Dissemination and Implementation (D&I) science Knowledge Translation (KT) Technical and professional communication Strategic scaling Marketing and commercialization ^c^ Public communication and stakeholder engagement (also related to community engagement and partnerships mentioned earlier)	Generates stakeholder feedback that informs continued development and implementation. Facilitates adoption, scaling, and sustained implementation of effective intervention programs. Supports continued local evaluation and broader scientific understanding of the intervention program.

***Notes*.** The examples provided within each column are intended to be illustrative rather than exhaustive. ^a^ Some, but not all, of the scholarship listed pertains specifically to school-based intervention work. Consequently, intervention scientists often draw upon multiple disciplines and collaborate with multidisciplinary teams to support high-quality intervention work. ^b^ *Community engagement* and *partnerships* is used broadly to encompass several related approaches, including community-engaged scholarship (CES), Community-Based Participatory Research (CBPR), Research-Practice Partnerships (RPPs), Participatory Action Research (PAR), Youth PAR, and other related approaches. ^c^ Marketing and commercialization does not necessarily imply financial profit. Rather, these activities may also support efforts to make effective intervention programs broadly available at low or no cost (see, for example, Begeny et al., 2011).

**Table 2:** Functional Conceptualization of the Four Domains of Intervention Work.

Domain	Primary Goal	Principal Products	Key Relevance for Intervention Scientists	Key Relevance for Intervention Adopters
**Development**	Create a high-quality intervention program	Theory-informed, manualized intervention program ready for evaluation	Guides program design, iterative refinement, and development of implementation supports	Helps determine whether a program was intentionally and comprehensively developed
**Implementation**	Understand how the program can be delivered successfully, taking into consideration diverse school-based contexts	Knowledge about feasibility, fidelity, adaptations, and implementation supports	Improves implementation quality and informs future development and evaluation	Helps determine whether the program is practical and sustainable in the adopter’s local context
**Evaluation**	Determine program quality, effectiveness, and appropriate use	Evidence regarding outcomes, implementation, and contexts for successful use	Supports refinement, scientific knowledge, and future research	Supports informed adoption decisions and ongoing program evaluation within the adopter’s school-based context
**Dissemination**	Increase awareness, access, adoption, and sustained use	Broader scientific knowledge, educational improvement, and societal impact	Facilitates scaling, knowledge translation, and broader scientific impact	Facilitates awareness, adoption, and long-term use of effective intervention programs

## Data Availability

No new data were created or analyzed in this study. Data sharing is not applicable to this article.

## Notes

1 Consistent with our professional research and practice, our aim for this paper is to integrate recommendations and scholarship that have relevance and implications internationally. However, because most of our work has taken place within school-based settings in the United States, readers will see that several of our examples or citations are derived from intervention work taking place within the United States. For a deeper consideration of how many of the core ideas of our article connect to internationality or internationalization, we refer interested readers to Begeny (2018), Begeny et al. (2024a), and van de Vijver (2013).

2 Although it is not the purpose of this paper to review existing frameworks for intervention development in detail, interested readers may wish to consult the NIH Stage Model (Onken, 2022; Onken et al., 2014), which provides one useful example of a comprehensive intervention framework.

3 In the context of assessing fidelity of individuals who deliver an intervention, we are aware that within much of the scholarship in implementation science that the term *intervention fidelity* is sometimes preferred over *implementation fidelity*. In this paper, we chose to use *implementation fidelity*, but *intervention fidelity* is conceptually synonymous.

4 The HELPS Program, mentioned earlier, offers one school-based illustration of several considerations described in this subsection. See, for example, Begeny et al. (2024b) and Durling et al. (2025) for extended discussion.

## Abbreviations

The following abbreviations are used in this manuscript:

MTSS	Multi-Tiered Systems of Support
D&I	Dissemination and Implementation

## Appendix A. Reflection Guide for Intervention Scientists

**Who is this Reflection Guide intended to benefit?**

We use the term *intervention scientist* to refer to researchers engaged in (or aspiring to engage in) one or more aspects of intervention program development, implementation, evaluation, or dissemination.

**How to Benefit from and Use this Reflection Guide:**

This Reflection Guide is intended to support professional judgment, collaborative planning, informed decision-making, and periodic self-assessment across the four domains of intervention work. Use the questions at major decision points and revisit them when new evidence, implementation experiences, or contextual circumstances warrant further reflection; they are prompts for thoughtful judgment rather than a scored or exhaustive checklist.

*To maximize use of this Reflection Guide, please also refer to the journal article in which the Guide was published.*

**Intervention Program Development**

1. **Need and Knowledge:** Is the educational need sufficiently important and clearly defined, and does a thorough examination of existing research, programs, strategies, theory, and relevant cultural or international scholarship support the need for this intervention program?
2. **Collaborative and Context-Responsive Design:** How have intended interventionists, recipients, families, school partners, and other stakeholders contributed to the development of the intervention’s goals, procedures, materials, and supports? How does the resulting design respond to authentic educational conditions and promote educational equity?
3. **Program and Manual Completeness:** Are the intervention’s core components, adaptable elements, procedures, materials, training and coaching supports, fidelity expectations, and local evaluation guidance sufficiently developed and tested? Does the program manual clearly document these and other important features, and does it provide practical guidance for high-quality implementation in authentic educational settings?
4. **Readiness and Stability:** Has the intervention reached sufficient stability for the proposed implementation, evaluation, or dissemination activities, and are the necessary expertise, partnerships, resources, and timelines available to complete any remaining development work responsibly?

**Intervention Program Implementation**

1. **Implementation in Practice:** How will usability, feasibility, acceptability, appropriateness, social validity, and equitable use be examined across authentic educational settings, interventionists, and recipients?
2. **Training and Continuing Support:** What knowledge, skills, training, coaching, feedback, and ongoing assistance do intended interventionists need, and are these supports both effective and feasible under typical educational conditions?
3. **Fidelity and Adaptation:** Are the intervention’s core components, permissible adaptations, and relevant dimensions of fidelity clearly defined, and how will implementation quality, dosage, participant responsiveness, and contextual influences be assessed?
4. **Learning from Implementation:** How will implementation experiences and data help distinguish among limitations of the program, inadequate implementation, insufficient support, inappropriate adaptations, and poor contextual fit? How will this knowledge strengthen subsequent development, evaluation, and dissemination?

**Intervention Program Evaluation**

1. **Evaluation Priorities:** Do the evaluation questions, outcomes, and measures align with the intervention’s educational purpose, theory, intended mechanisms, and stage of maturity while retaining meaningful intervention effects as a central focus?
2. **Effects and Variation:** Does evaluation provide sufficient implementation information to interpret outcomes and examine how effects are produced and whether they vary across recipients, interventionists, settings, implementation conditions, and historically underserved populations?
3. **Credible Evidence:** Which quantitative, qualitative, or mixed methods are needed to answer the most important evaluation questions? Across what populations, interventionists, settings, implementation conditions, and research teams—including teams with little or no involvement from the intervention developers—should findings be replicated before broader conclusions are warranted?
4. **Responsible Conclusions and Continued Evaluation:** Do claims about effectiveness, generalizability, evidence-based status, and readiness for adoption reflect the strength and scope of the available evidence? Does the intervention program provide feasible guidance and resources that enable adopters to conduct meaningful local evaluation in the school-based settings where it is implemented?

**Intervention Program Dissemination**

1. **Readiness for Practice Dissemination:** Is the intervention sufficiently developed, stable, implementable, and supported by evidence for broader school-based use with substantially less direct involvement from its developers?
2. **Information and Resources:** Do the program manual and related resources communicate the intervention’s purpose, evidence, limitations, intended users and recipients, implementation requirements, fidelity expectations, permissible adaptations, training supports, and local evaluation guidance clearly and accurately?
3. **Access and Adoption:** How will dissemination models, communication strategies, costs, copyright or licensing arrangements, organizational partnerships, and support structures promote equitable access, evidence-driven adoption, and responsible use?
4. **Scaling and Stewardship:** What evidence, infrastructure, partnerships, and quality-assurance systems are needed before strategic scaling? Who will remain responsible for technical assistance, continued evaluation, program integrity, version control, communication of new evidence, and future revisions?

## Appendix B. Reflection Guide for Intervention Adopters

**Who is this Reflection Guide intended to benefit?**

We use the term *intervention adopters* to refer to individuals or teams responsible for selecting whether to adopt and use school-based intervention programs within a particular educational context. These may include classroom educators, school principals, district curriculum directors, Multi-Tiered Systems of Support (MTSS) leadership teams, afterschool program directors, district-level committees, support staff, and others.

**How to Benefit from and Use this Reflection Guide:**

This Reflection Guide is intended to support informed intervention selection, implementation planning, local decision-making, collaborative discussion, and periodic self-assessment. Use the questions before adoption, during implementation, and when reviewing continued use; they are prompts for professional judgment rather than a scored or exhaustive checklist. If you cannot answer some of the questions, it is recommended that you make attempts to communicate with the intervention program developer(s) and/or the individuals responsible for making the intervention program available to potential adopters and users.

*To maximize use of this Reflection Guide, please also refer to the journal article in which the Guide was published.*

**Intervention Program Development**

1. **Need and Knowledge:** Does the intervention address a clearly defined and locally important educational need, and what research, theory, existing programs or strategies, and stakeholder knowledge informed its development?
2. **People and Contexts:** Were interventionists, recipients, families, schools, and communities resembling ours meaningfully represented during development, and does the program account for our students, personnel, systems, resources, and cultural and linguistic context?
3. **Program Completeness:** Are the intervention procedures, core components, adaptable elements, materials, program manual, training and coaching supports, fidelity expectations, and local evaluation resources sufficiently clear and complete for responsible use?
4. **Maturity and Stability:** Is the program sufficiently developed, tested, and stable for our intended purpose, and are its developmental status, evidence, revision history, limitations, and any remaining uncertainties communicated transparently by those disseminating the program?

**Intervention Program Implementation**

1. **Local Feasibility:** Is the intervention usable, feasible, socially valid, and equitable under our actual conditions, including staffing, schedules, technology, leadership, resources, and existing systems of support?
2. **Personnel and Support:** Who will implement the intervention, and do those individuals have the necessary knowledge, skills, time, training, coaching, feedback, and opportunities for assistance to implement the program with appropriate quality and fidelity?
3. **Fidelity and Adaptation:** Which intervention components must remain consistent; what adaptations are permissible; and how will implementation quality, dosage, fidelity, interventionist experience, recipient responsiveness, and barriers be monitored?
4. **Responding to Implementation Findings:** If challenges or disappointing outcomes occur, can we determine whether they reflect inadequate implementation, insufficient support, an inappropriate adaptation, poor contextual fit, or a limitation of the program? Does the program manual provide sufficient guidance for understanding what should occur when the intervention is implemented appropriately?

**Intervention Program Evaluation**

1. **Relevance of the Evidence:** What meaningful outcomes has the intervention been shown to produce, and how closely do the studied goals, recipients, interventionists, settings, and implementation conditions resemble our own?
2. **Strength of the Evidence:** How rigorous, consistent, and replicated is the available evidence, and what do claims that the intervention is *effective* or *evidence-based* actually mean?
3. **Local Evaluation:** How will we examine both implementation and meaningful outcomes in our setting, including feasibility, equitable access, differential outcomes, unintended effects, and the perspectives of interventionists and recipients?
4. **Decision Criteria:** What research evidence, local outcomes, implementation findings, contextual information, and stakeholder perspectives would support maintaining, expanding, adapting, reducing, or discontinuing use of the intervention?

**Intervention Program Dissemination**

1. **Information for Adoption:** Are the intervention’s purpose, evidence, limitations, appropriate populations, required conditions, and current level of readiness for broader use communicated clearly and accurately?
2. **Access and Support:** Are the program manual, materials, training, coaching, technical assistance, fidelity guidance, adaptation guidance, and evaluation resources accessible and sufficient, and could financial, technological, staffing, licensing, or geographic barriers undermine equitable or sustained use?
3. **Evidence-Driven Decision-Making:** Is the adoption decision based primarily on credible evidence of program value and local fit rather than popularity, persuasive marketing, institutional habit, political pressure, prior financial investment, misleading information, misinformation, or unsupported claims?
4. **Ongoing Stewardship:** Does the organization disseminating the intervention have the expertise, infrastructure, and incentives needed to support high-quality use, and how will users receive technical assistance, revised materials when warranted, program updates, and new research findings over time?
